# Atypical Presentation of Herpes Stromal Keratitis in a Contact Lens Wearer

**DOI:** 10.7759/cureus.38438

**Published:** 2023-05-02

**Authors:** Ruknesvary Subramaniam, Khairy Shamel Sonny Teo, Julieana Muhammed

**Affiliations:** 1 Department of Ophthalmology and Visual Sciences, School of Medical Sciences, Universiti Sains Malaysia, Kubang Kerian, MYS

**Keywords:** ring infiltrate, herpes simplex virus, acanthamoeba keratitis, herpes simplex keratitis, herpes stromal keratitis

## Abstract

This article describes the case of a 21-year-old female habitual contact lens wearer who complained of left eye pain, redness, and decreased vision for one week. When a ring-shaped corneal infiltration indicative of an Acanthamoeba infection was discovered, standard anti-amoebic topical therapy with polyhexamethylene biguanide and chlorhexidine was commenced. However, her keratitis worsened. At the same time, corneal scraping revealed no pathogens. An anterior chamber examination revealed a loss of corneal sensation, and a positive herpes simplex virus (HSV) immunoglobulin G serology test indicated HSV keratitis. She was eventually treated with oral anti-viral medication and recovered completely. Her case was unusual, as she had a history of contact lens use, painful corneal ulceration, and the development of Acanthamoeba keratitis-like corneal ring infiltration. This case also reinforces the various manifestations of HSV keratitis, which lead to delayed diagnosis and treatment.

## Introduction

Corneal ulceration is an inflammatory or infectious disorder of the cornea that involves the rupture of the epithelial layer and the involvement of the cornea [1]. Infectious corneal ulcers are the fourth most common cause of blindness in Malaysia, according to the results of the National Eye Survey, which was carried out there in 1996 [2].

Herpes simplex virus (HSV) is frequently identified as a clinical masquerader due to its potentially perplexing and varied appearance. These variances can cause a diagnosis delay, which affects the patient's quality of life and overall visual outcomes. The problem of treating HSV ocular infections has gained significance because of their devastating corneal symptoms and the high seroprevalence of these viruses in the community [3]. As a result, herpes stromal keratitis (HSK) caused by HSV is listed among the leading causes of infectious corneal blindness in developed countries [4,5]. The involvement of the corneal stroma typically manifests as stromal infiltrates, edema, and neovascularization. HSV can be challenging to diagnose, especially when it manifests unusually. On the other hand, Acanthamoeba corneal infections are frequently reported in contact lens (CL) wearers, especially among those with poor CL-handling techniques [6]. This case report describes a rare instance of HSV-related corneal infection in a young CL wearer who first presented with corneal ring infiltrates that seemed like Acanthamoeba keratitis (AK).

“This article was previously presented as a meeting abstract at the 4TH USIM International Health E-Conference In Conjunction With The 3rd International Conference On Medicine And Health Science (IHEC) on 16-17 December 2020.”

## Case presentation

History

A 21-year-old female who regularly wears soft CLs presented to our ophthalmology clinic with a complaint of left eye (LE) pain, redness, and reduced vision for one week. She gave a history of wearing CLs for 10 days preceding the onset of the symptoms. The solution that she used to clean her CL was first opened more than four months ago. She had not been swimming in her CLs or used tap water to clean them, and she had no complaints about her right eye (RE). Her past ocular history was significant for the daily wearing of soft CLs for over six years. She had no medical history or any history of trauma. She was a nonsmoker and denied any allergies to medications. She reported no history of ocular disease or eye surgery.

Examination

**Figure 1:**
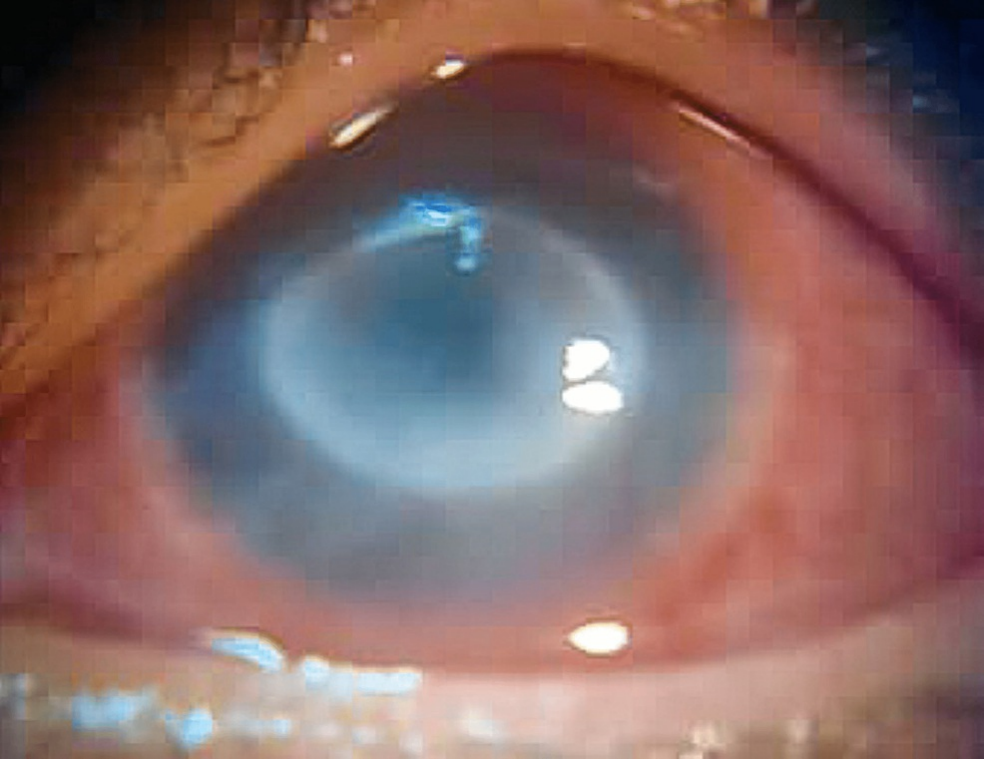
Initial presentation of a ring-like stromal infiltrate and a lack of bulbous dendrites

The best-corrected visual acuities of her LE and RE were 20/80 and 20/20, respectively. Ocular examination using slit-lamp biomicroscopy revealed a central corneal anterior stroma ring infiltrate with an irregular border. It was associated with an edematous cornea (Figure 1). The conjunctiva was moderately injected. There was no hypopyon, but a minimal anterior chamber reaction was found. The intraocular pressure in both eyes was measured to be 14 mm Hg using a non-contact tonometer. A funduscopic examination did not result in anything remarkable.

Management

**Figure 2:**
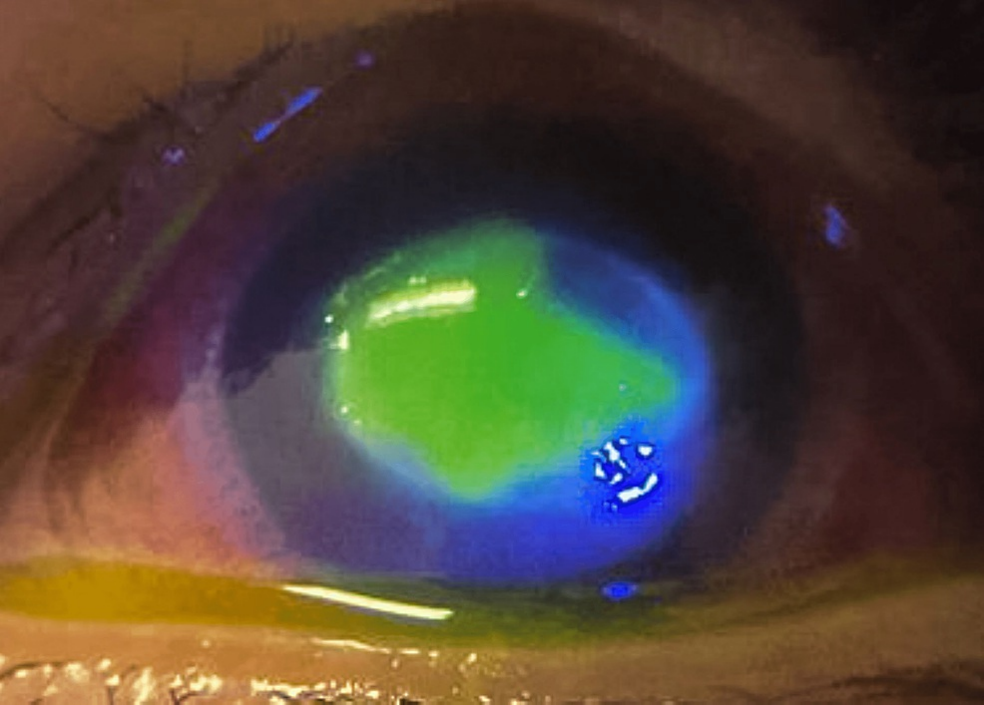
Worsening infiltrates with a persistent epithelial defect

She was treated for AK with a combination of topical polyhexamethylene biguanide four times a day, and we commenced with chlorhexidine (0.02%) every two hours. However, she did not show any improvement. Her vision subsequently deteriorated, and the ring infiltrates extended into the posterior corneal stroma with worsening corneal edema. A persistent epithelial defect was present, which was confirmed with corneal fluorescein staining (Figure 2).

Investigation

Before the initiation of the eyedrops, corneal scrapings were taken for microscopy, culture, and sensitivity tests. Initial microscopic examination and culture tests did not show any growth. A CL solution for Acanthamoeba sp. polymerase chain reaction (PCR) also produced negative results. Because the patient failed to respond to standard therapy for AK and the culture of the cornea for Acanthamoeba was negative, we had second thoughts about the diagnosis. Further examination showed a decrease in LE corneal sensation, which was previously intact. Additional serology tests confirmed the presence of HSV immunoglobulin G. The patient’s anti-Acanthamoeba treatment was discontinued, and we commenced with oral Acyclovir 800 mg five times per day.

Outcome

Over the next several days, the ulcer gradually responded to the treatment, and the patient felt more comfortable. Ocular examinations showed substantial improvements in the presence of corneal stromal scarring and the diminution of corneal edema. Five months after the initial presentation, the best corrected visual acuity of the patient’s LE was 20/100.

## Discussion

This unique case serves as further evidence that Acanthamoeba is unquestionably an excellent mimic of keratitis, as some well-known studies have shown. The patient was initially diagnosed with AK as she demonstrated classical findings associated with this disease, including the presence of eye pain, a clinical presentation of ring-shaped corneal stroma infiltrates, and the most important risk factor-a history of wearing CLs [7].

The revision of our initial diagnosis was necessary when the patient failed to respond to the standard treatment given, and the cultures were negative for Acanthamoeba. The ring-shaped infiltrates seen in this patient are caused by an immune response involving an antigen-antibody complex formation called the Wessely stromal immune ring [8]. It must be emphasized that the presence of stromal ring-shaped infiltrates is not exclusively associated with Acanthamoeba infection. It is also commonly seen in other infectious and non-infectious agents, namely HSV and the chronic wearing of CLs, respectively [9], as seen in our case.

At present, HSV is diagnosed by its clinical presentation on a slit-lamp examination, but the literature does not always support the accuracy of those clinical findings. Several diagnostic tests, such as PCR assays, enzyme-linked immunosorbent assays, immunofluorescent antibodies, and viral cultures, have provided more definitive diagnoses, but they have some limitations [10]. Except for active cases of HSV corneal epithelial infection, which may be cultured and feature a distinct dendritiform appearance, there is no absolute test for diagnosing HSV disease.

As different pathogens might appear with comparable symptoms in clinical settings, misdiagnosis is not unusual. Studies have demonstrated that HSV keratitis has incorrectly been classified as both amebic and fungal diseases [11]. The diagnosis of HSK should be considered in patients with Acanthamoeba disease who respond poorly to the appropriate standard therapy.

## Conclusions

The atypical presentation of HSK presents a diagnostic challenge and may mistakenly be diagnosed as AK, especially in patients with a history of CL use. While it is known that Acanthamoeba may masquerade as HSV keratitis, especially in its early stages, an Acanthamoeba-like presentation of HSV keratitis has only been reported once in the existing literature. Nevertheless, early detection and treatment are crucial to preventing long-term vision impairment.
